# Rhinogenic optic neuropathy with hemianopia caused by ethmoidal sinus mucocele

**DOI:** 10.1002/ccr3.6696

**Published:** 2022-12-05

**Authors:** Kosuke Oka, Wataru Ando, Hideharu Hagiya, Takaya Higaki, Fumio Otsuka

**Affiliations:** ^1^ Department of General Medicine Okayama University Graduate School of Medicine, Dentistry and Pharmaceutical Sciences Okayama Japan; ^2^ Okayama University Medical School Okayama Japan; ^3^ Department of Otolaryngology‐Head & Neck Surgery Okayama University Graduate School of Medicine, Dentistry and Pharmaceutical Sciences Okayama Japan

**Keywords:** rhinogenic optic neuropathy

## Abstract

A patient complained of acute right vision loss and headache. A computed tomography scan revealed ethmoidal sinus mucocele in the right ethmoid sinus that was compressing the optic nerve and emergency endoscopic sinus surgery was performed.

## Graphical abstract

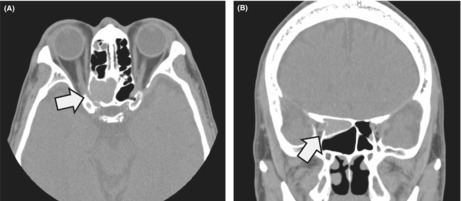

A 61‐year‐old man without a particular medical history complained of acute right vision loss and headache. The visual field of the right eye showed temporal hemianopia. Tenderness was noted in the right eye, and the right eyelid was swollen. The right eyelid conjunctiva was hyperemic, but there was no abnormal eye movement or pupillary irregularity. A computed tomography scan revealed mucosal thickening and liquid retention in the right ethmoid sinus with accompanying destruction of the adjacent bone and compression of the optic nerve (Figure 1A: axial, arrow and Figure 1B: coronal, arrow). Emergency endoscopic sinus surgery was performed, but the visual loss did not recover completely.

**FIGURE 1:**
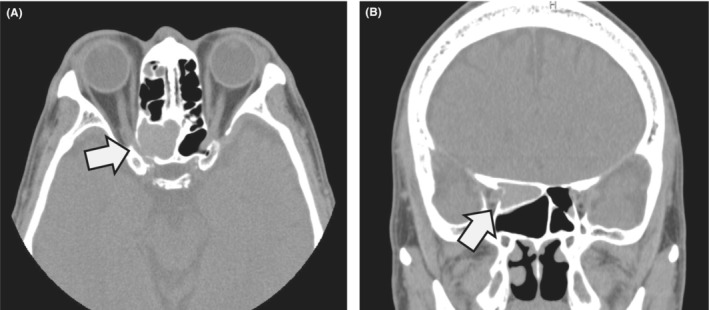
Paranasal cavity CT scan. Mucosal thickening and liquid retention in the right ethmoid sinus with accompanying destruction of the adjacent bone and compression of the optic nerve (A: axial, arrow and B: coronal, arrow).

Rhinogenic optic neuropathy should be considered in patients with acute vision loss, especially when unilateral temporal hemianopia is observed.^1^ Early diagnosis and surgical intervention can help to prevent the complication of permanent blindness due to optic nerve injury.^2^

## AUTHOR CONTRIBUTIONS

**Kosuke Oka:** Supervision; writing – original draft; writing – review and editing. **Wataru Ando:** Writing – original draft. **Hideharu Hagiya:** Supervision. **Takaya Higaki:** Supervision. **Fumio Otsuka:** Supervision.

## FUNDING INFORMATION

None.

## CONFLICT OF INTEREST

The authors declare no conflicts of interest.

## CONSENT

Written informed consent was obtained from the patient to publish this case report.

## Data Availability

The datasets during the current paper are available from the corresponding author on reasonable request.
